# Skin dysbiosis in the microbiome in atopic dermatitis is site-specific and involves bacteria, fungus and virus

**DOI:** 10.1186/s12866-021-02302-2

**Published:** 2021-09-23

**Authors:** Rie Dybboe Bjerre, Jacob Bak Holm, Albert Palleja, Julie Sølberg, Lone Skov, Jeanne Duus Johansen

**Affiliations:** 1 National Allergy Research Centre, Herlev and Gentofte Hospital, University of Copenhagen, Copenhagen, Denmark; 2 Clinical Microbiomics, Fruebjergvej 3, 2100 Copenhagen, Denmark; 3 Department of Dermatology and Allergy, Herlev and Gentofte Hospital, University of Copenhagen, Copenhagen, Denmark

**Keywords:** Atopic dermatitis, Skin microbiome, Dysbiosis

## Abstract

**Background:**

Microbial dysbiosis with increased *Staphylococcus aureus* (*S. aureus*) colonization on the skin is a hallmark of atopic dermatitis (AD), however most microbiome studies focus on bacteria in the flexures and the microbial composition at other body sites have not been studied systematically.

**Objectives:**

The aim of the study is to characterize the skin microbiome, including bacteria, fungi and virus, at different body sites in relation to AD, lesional state, and *S. aureus* colonization, and to test whether the nares could be a reservoir for *S. aureus* strain colonization.

**Methods:**

Using shotgun metagenomics we characterized microbial compositions from 14 well defined skin sites from 10 patients with AD and 5 healthy controls.

**Results:**

We found clear differences in microbial composition between AD and controls at multiple skin sites, most pronounced on the flexures and neck. The flexures exhibited lower alpha-diversity and were colonized by *S. aureus*, accompanied by *S. epidermidis* in lesions. *Malassezia* species were absent on the neck in AD. Virus mostly constituted *Propionibacterium* and *Staphylococcus* *phages*, with increased abundance of *Propionibacterium phages PHL041* and *PHL092* and *Staphylococcus epidermidis phages CNPH82* and *PH15* in AD. In lesional samples, both the genus *Staphylococcus* and *Staphylococcus phages* were more abundant. *S. aureus* abundance was higher across all skin sites except from the feet. In samples where *S. aureus* was highly abundant, lower abundances of *S. hominis* and *Cutibacterium acnes* were observed. *M. osloensis* and *M. luteus* were more abundant in AD. By single nucleotide variant analysis of *S. aureus* we found strains to be subject specific. On skin sites some *S. aureus* strains were similar and some dissimilar to the ones in the nares.

**Conclusions:**

Our data indicate a global and site-specific dysbiosis in AD, involving both bacteria, fungus and virus. When defining targeted treatment clinicians should both consider the individual and skin site and future research into potential crosstalk between microbiota in AD yields high potential.

**Supplementary Information:**

The online version contains supplementary material available at 10.1186/s12866-021-02302-2.

## Background

The human skin is colonized by a variety of microorganism, interacting with the host and modulating immunity. On healthy human skin, the most abundant bacterial genera are *Cutibacterium*, *Staphylococcus* and *Corynebacterium* with marked topographical diversity [1].

In the common skin disease atopic dermatitis (AD) [2], *Staphylococcus aureus* expand and conventional culture-based studies [3] find colonizing frequencies of 70% of lesional and 39% of non-lesional sites, and 62% of the nares samples [4]. *S. aureus* colonization adversely affect disease severity [5]. In recent years, skin microbiomes in AD have been studied in a variety of conditions [5–10]. Most studies are based on sequencing the 16S rRNA gene of bacteria. Applying this method, bacterial diversity has been shown to be lower in AD [5, 7, 11] and *S. epidermidis* abundant [5, 12]. Therapy increases diversity [9] and the abundances of *Streptococcus*, *Cutibacterium* and *Corynebacterium* [5]. By applying shotgun sequencing of whole metagenomes a better taxonomical resolution is achieved and all domains can be analysed. Studies applying this method in AD are emerging [8, 10, 13, 14] and describe specific *S. aureus* strains in severe AD [8], perturbations in the eukaryotic community [13], and define AD subgroups [14].

There is growing evidence of a key role of the microbiome in the pathogenesis of AD [15]. This is supported by studies showing that microbiome dysbiosis can precede AD in early childhood [16, 17]. Though there might be a critical window for establishing a healthy microbiome and immune tolerance toward it in early childhood [18], studies applying topical commensals to re-establish a healthy microbiome in AD show improvements in the disease [19–21]. However, benefits of using commensals have been reported to be dependent on skin site, for instance with a treating effect of transplanting *R. mucosa* in the antecubital flexure of AD patients but no effect on hands [19]. In general, most microbiome studies in AD focus on the body flexures but do not address microbial composition at other body sites. Furthermore, virus in AD has not been well investigated. Here, we present a case-control study applying shotgun metagenomics to characterize the skin microbiome of AD patients at different body sites.

## Results

Samples from 5 healthy controls (3 women, 2 men), aged 27–63 and 10 patients with AD (7 women and 3 men), aged 24–62 years, were included in this study. Mean Severity Scoring of Atopic Dermatitis (SCORAD) for patients with AD was 30.8 (Table 1). Of 212 samples (including *E. coli* and buffer controls), 91 samples were of insufficient DNA quality and/or amount for sequencing (Table 1). Success of library preparation in lesional samples were 45% (32/71), 39% (27/69) in non-lesional and 87% (61/70) in controls. Other factors influencing success of library preparation were related to subject and skin site (Table 1).

**Table 1:** Characterization of the study population and samples

Characteristics	Atopic dermatitis	Healthy controls
Subjects analysed, N	10	5
Age, mean (range), years	47 (24–62)	48 (27–63)
Female:male ratio	7:3	3:2
SCORAD		
Mean (range)	31 (20–68)	NA
Moderate: Severe	8:2	NA
Filaggrin Mutation: Wt: Unknown	4:1:5	Unknown
HECSI, mean (range)	10 (4–16)	NA
Treatment		
No	2	5
Steroid	5	0
Systemic	4	0
Nizoral		
Occasionally: likely after study participation	1:1	0
Co-morbidities		
Asthma	5	0
Hay fever	5	0
CD	5	1
FA	1	0
**Skin site successfully sampled, Nonlesional:lesional ratio**		
Nasal	8:1	5
Periorbital	2:3	5
Perioral	4:4	5
Neck	2:3	5
Upper inner arms	0:2	3
Antecubital fossae	1:3	5
Volar forearms	0:2	3
Dorsum of hands	0:1	3
Palmar hands	3:2	3
Between fingers	0:5	5
Popliteal flexures	1:1	4
Dorsum of feet	0:2	5
Arches of feet	0:1	5
Between toes	6:2	5

The 14 skin areas sampled are listed in the top of the table and in detail include: The neck (the anterior triangle), and bilaterally from the anterior nares, periorbital and perioral areas, antecubital and popliteal flexures (midline +/− 5 cm), upper inner arms (starting after the flexural area ending before the armpit, before presence of hair follicles from the armpit), volar forearms (starting after the antecubital fossae to 4 cm from the wrist), dorsum of the hands and feet (from wrist to joints of the digits), the web spaces between the fingers and toes, palmar hands (from wrist to joints of the digits), and arches of the feet.

*Abbreviations*: *AD* atopic dermatitis, *C* control, *M* male, *F* female, *SCORAD* Severity Scoring Atopic Dermatitis, *HECSI* Hand Eczema Severity Index, *FLG* filaggrin gene, *UN* unknown, *CD* contact dermatitis, *FA* food allergy, *WT* wildtype, *Mut* mutation, *NA* not applicable.

The sequencing generated an average of 36 million read pairs per sample. Initially, data were described according to the 14 skin sites sampled. When analyzing the effect of lesions, the 14 skin sites were pooled, with a minimum number of 5 samples per group.

### Beta diversity revealed characteristic AD skin sites

**Fig. 1:**
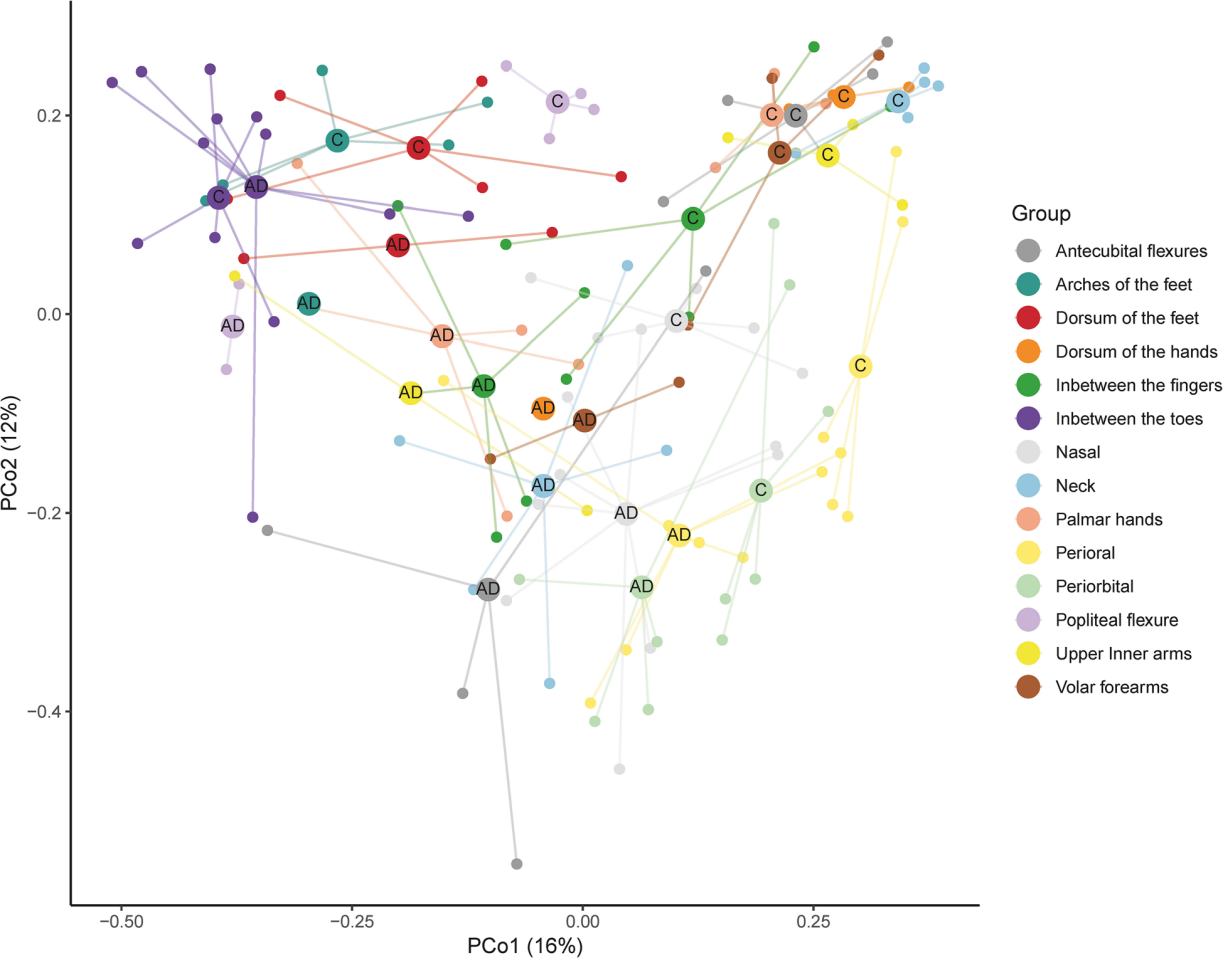
Characteristic AD skin sites. Principal coordinate analysis plot based on Bray-Curtis distances between healthy control and AD samples within each skin site. Centroids represent the arithmetic mean position of the points belonging to the specific category. Samples from the hands, arms, and flexures separate according to AD, whereas feet, periorbital and perioral areas do not

Subject explained the majority of the explained microbial variance (PERMANOVA test; *R*^2^ = 19%; *P* = 0.0001) (Fig. S11), however, the overall skin microbial composition differed significantly between AD and controls (PERMANOVA test; *R*^2^ = 6%, *P =* 0.0001). As visualized on the principal coordinate analysis (PCoA, Fig. 1), samples from the hands and arms, flexures and neck showed the clearest separation according to control or AD (Fig. S1). The lowest separation was observed for perioral and periorbital samples.

### Alpha diversity and bacterial species in AD and healthy controls

**Fig. 2:**
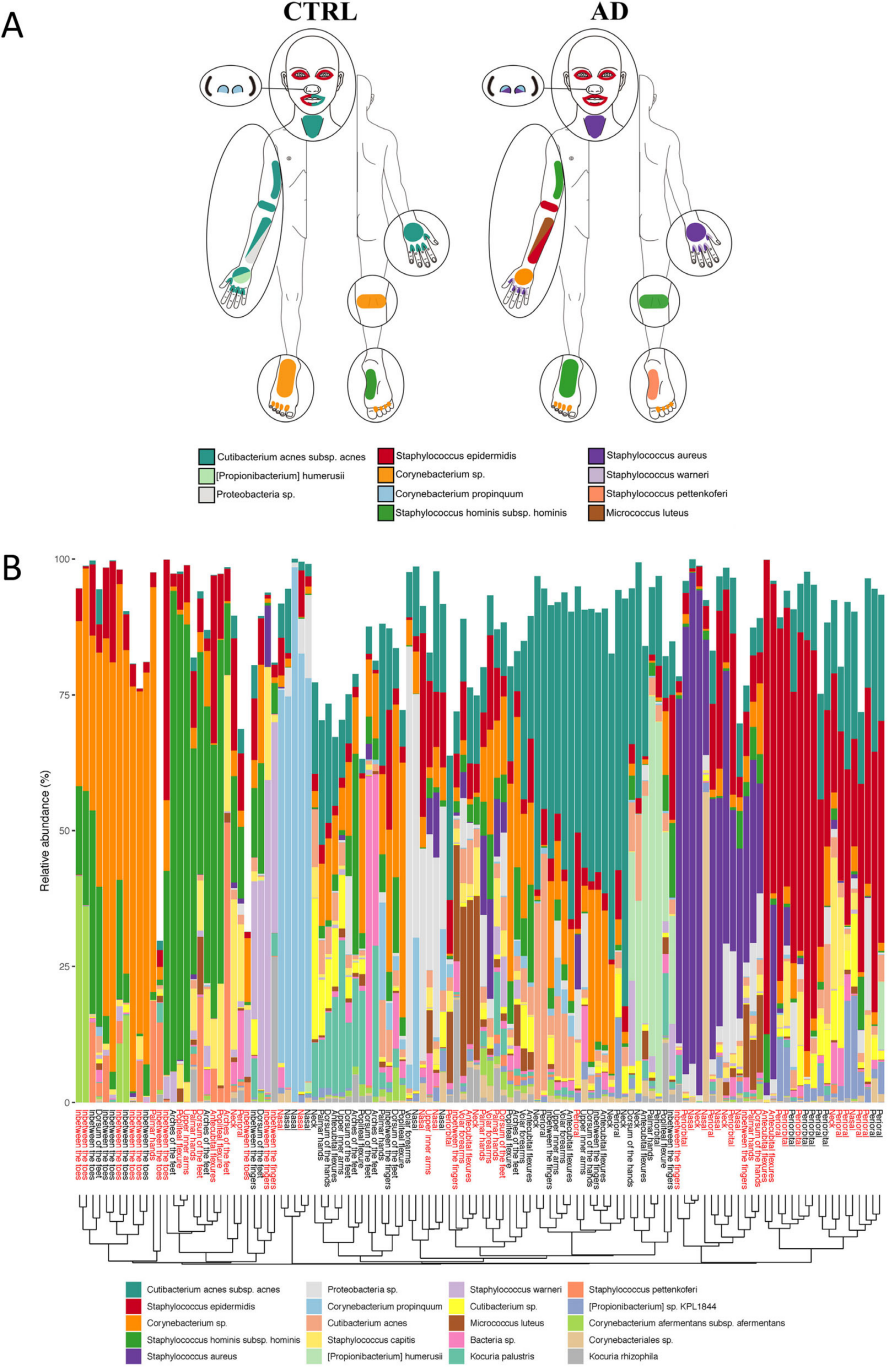
Bacterial species at different skin sites in healthy controls and patients with AD. **A**, Illustration of the most predominant bacterial species at the 14 non-overlapping skin areas investigated: Two species are depicted at one skin site when the second most abundant specie was within 5% in total rel. Abundance compared to the most predominant. **B**, Stacked bar plots of the relative abundances of the 20 bacterial taxa with highest average abundances across all samples, arranged according to similarity (Bray-Curtis). Skin site is stated in red for AD and black for healthy control samples

Initial exploration of differences in the microbiome composition showed lower bacterial alpha-diversity at the flexures in AD (Fig. S2). The flexures in AD were dominated by the genus *Staphylococcus,* mostly the species *S. epidermidis* and *S. aureus* (Fig. 2 and S3).

*S. aureus* was low or undetected in control samples but present at most skin sites among AD patients and occasionally dominated the community (Fig. S3). Individual differences were also seen in *S. aureus* colonization, where AD10 was highly colonized across all skin sites (except from the feet, Fig. S4). Other species more abundant in AD included *M. luteus, S. epidermidis, S. saccharolyticus, S. lugdunensis, M. osloensis* and *Rothia sp. ND6WE1A* (Fig. 2 and Table 2). On the contrary species higher in abundance in controls include *Cutibacterium acnes, [Propionibacterium] humerusii, Corynebacterium sp.* and *Corynebacterium singular* (Figs. 2, S3 and Table 2).

**Table 2:** Low abundant species and their presence in control and AD patients at different skin sites

	Pos. individuals			Mean relative abundance (%)																											
	(> 0% at one site)/total			Nasal		Periorbital		Perioral		Neck	Upper inner arms			Antecubital flexures		Volar forearms		Dorsum of hands		Palmar hands		Between fingers		Popliteal flexures		Dorsum of feet		Arches of feet		Between toes	
**Species**	**C**	**AD**	**Subjects with site(s) ≥ 1%**	**C**	**AD**	**C**	**AD**	**C**	**AD**	**C**	**AD**	**C**	**AD**	**C**	**AD**	**C**	**AD**	**C**	**AD**	**C**	**AD**	**C**	**AD**	**C**	**AD**	**C**	**AD**	**C**	**AD**	**C**	**AD**
*M. osloensis*	5/5	9/9	AD2, AD3, AD4, AD8, AD9	0.0	0.1	0.0	0.2	0.0	0.1	0.0	0.5	0.2	1.6	0.1	0.4	0.1	1.5	0.1	1.2	0.1	0.8	0.1	2.7	0.2	0.6	0.2	0.4	0.2	0.1	0.0	2.4
*P. yeei*	5/5	7/9	AD3	0.0	0.0	0.0	2.0	0.0	0.2	0.0	0.2	0.2	0.1	0.2	1.5	0.0	0.3	0.3	0.1	0.0	0.0	0.0	2.4	0.2	0.0	0.0	0.1	0.0	0.0	0.0	0.6
*Rothia sp. ND6WE1A*	0/5	3/9	AD9	0.0	0.2	0.0	0.0	0.0	0.8	0.0	0.3	0.0	0.0	0.0	0.0	0.0	0.0	0.0	0.0	0.0	0.5	0.0	0.5	0.0	1.1	0.0	0.1	0.0	0.1	0.0	0.0
*Malassezia globosa*	5/5	8/9	C5, C6, C8, C9, C10, AD2, AD6	0.0	0.4	0.1	0.2	0.5	0.3	2.0	0.2	0.4	0.1	1.3	0.1	1.6	0.2	0.3	0.0	0.3	0.2	0.2	0.1	0.1	0.0	0.1	0.2	0.1	0.0	0.0	0.0
*Malasseziales sp.*	5/5	9/9	C5, C6, C8, C9, C10, AD6, AD8	0.0	0.0	0.1	0.0	0.0	0.0	1.1	0.0	0.1	0.0	0.1	0.0	0.1	0.0	0.2	0.1	0.0	0.0	0.1	0.0	0.0	0.0	0.0	0.0	0.0	0.0	0.0	0.0
*Staphylococcus saccharolyticus*	1/5	4/9	AD2, AD3	0.0	0.0	0.0	3.9	0.0	0.2	0.0	3.4	0.1	0.0	0.0	1.1	0.0	0.7	0.1	0.0	0.0	0.0	0.0	0.4	0.0	0.0	0.0	0.0	0.0	0.0	0.0	0.0
*Staphylococcus lugdunensis*	2/5	7/9	AD9	0.0	2.6	0.0	0.0	0.0	0.2	0.0	0.8	0.0	0.2	0.0	0.3	0.0	0.0	0.0	0.0	0.0	0.2	0.0	0.1	0.0	0.1	0.0	0.3	0.0	0.1	0.0	0.0
*Staphylococcus cohnii*	4/5	4/9	C9, C10	0.0	0.0	0.0	0.0	0.0	0.0	0.0	0.0	0.0	0.0	0.0	0.0	0.0	0.0	0.1	0.0	0.0	0.0	0.0	0.0	0.2	0.0	0.6	0.1	2.5	0.0	0.0	0.0
*Staphylococcus pasteuri*	5/5	8/9	C5, C6, AD1, AD2, AD3, AD9	0.0	0.1	0.1	0.3	0.0	0.3	0.1	0.7	0.2	0.0	0.2	0.5	0.1	0.4	0.5	0.1	0.8	1.0	2.6	2.8	0.2	0.0	0.3	0.5	0.1	0.1	0.0	0.1
*Staphylococcus haemolyticus*	5/5	7/9	C5, C9, C10, AD1, AD9, AD10	0.0	0.1	0.0	0.1	0.0	0.2	0.0	0.3	0.1	0.5	0.1	1.1	0.0	0.2	0.5	0.3	0.4	0.6	0.3	0.2	0.8	0.2	1.7	2.6	0.6	1.0	0.1	8.1
*C. singulare*	4/5	4/9	C6	0.1	0.0	0.2	0.0	0.0	0.0	0.1	0.0	0.3	0.1	0.4	0.0	0.0	0.0	0.6	0.0	1.7	0.0	1.2	0.0	1.3	0.0	0.4	0.1	0.4	0.0	0.0	0.0
*C. appendicis*	2/5	1/9	C8	0.1	0.0	0.0	0.0	0.0	0.0	0.0	0.0	0.0	0.0	0.4	0.0	0.6	0.0	0.0	0.0	0.0	0.0	0.1	0.0	0.1	0.0	0.3	0.0	1.9	0.0	0.0	0.0
*C. jeikeium*	5/5	3/9	C9, AD8	0.0	0.0	0.0	0.2	0.0	0.1	0.0	0.1	0.2	0.1	0.3	0.0	0.1	0.3	0.1	0.4	0.6	0.3	0.4	0.1	1.6	0.0	0.4	0.5	0.2	0.0	0.1	1.7
*C. simulans*	4/5	6/9	C5, C6, AD8	0.1	0.1	0.0	0.0	0.0	0.0	0.0	0.0	0.0	0.0	0.0	0.0	0.0	0.0	0.0	0.1	0.1	0.0	0.0	0.0	0.4	0.0	0.1	0.0	0.0	0.0	0.5	0.2
*Streptococcus thermophilus*	5/5	8/9	C6, C9, AD4	0.1	0.0	1.9	0.1	1.1	0.3	0.1	0.1	1.0	0.2	1.9	0.0	0.3	0.2	0.8	0.1	1.5	0.2	0.5	0.1	0.4	0.0	0.4	0.0	0.2	0.0	0.0	0.0
*Streptococcus gordonii*	5/5	8/9	C6, C8, AD1, AD10	0.0	0.0	0.0	0.3	1.0	0.8	0.2	0.1	0.2	0.0	0.1	0.1	0.4	0.2	0.1	0.1	0.2	0.6	0.1	0.7	0.0	0.0	0.1	0.1	0.0	0.0	0.0	0.0
*Streptococcus oralis*	4/5	8/9	C6, C10, AD4, AD9, AD10	0.0	0.0	0.0	0.2	0.3	1.0	0.0	0.1	0.5	0.1	0.0	0.1	0.1	0.2	0.0	0.1	0.0	0.5	0.0	0.9	0.0	0.0	0.3	0.1	0.0	0.0	0.0	0.0
*K. marina*	5/5	8/9	C5, C6, AD9	0.3	0.0	0.8	0.2	0.7	0.1	1.9	0.1	3.2	0.0	2.5	0.2	0.4	0.3	2.5	0.0	2.0	0.3	0.7	0.3	3.1	0.0	1.5	0.1	2.1	0.0	0.0	0.3
*K. palustris*	5/5	9/9	AD1, AD4, AD9, AD10, C5, C6, C8, C9	0.1	0.1	0.4	0.3	0.2	0.4	2.0	0.2	9.5	0.1	4.5	0.1	0.6	0.4	5.0	0.3	4.2	3.2	2.0	2.3	7.5	0.0	3.2	2.5	9.0	0.0	0.1	0.5
*Kocuria sp. WRN011*	5/5	5/9	C5, C6, AD1, AD9, AD10	0.2	0.0	0.1	0.3	0.1	1.1	0.0	0.1	0.6	0.0	0.5	0.1	0.3	0.1	0.9	0.0	0.8	1.0	0.7	0.3	0.7	0.0	0.5	0.3	0.5	0.0	0.1	0.2
*Cutibacterium avidum*	5/5	7/9	C8, AD1, AD8	1.0	0.6	0.1	0.0	0.0	0.1	0.0	0.0	0.1	0.0	0.0	0.0	0.1	0.0	0.0	0.0	0.0	0.1	0.0	0.0	0.0	0.0	0.0	0.0	0.0	0.0	0.0	0.0
[*Propionibacterium*] *namnetense*	5/5	8/9	C5, C10, AD4, AD6, AD10	0.1	0.0	0.3	0.6	0.2	0.6	0.5	0.3	0.4	0.0	1.0	0.0	0.3	0.2	0.6	0.1	0.8	0.4	1.0	0.1	0.4	0.0	0.1	0.1	0.0	0.0	0.0	0.0
*Veillonella parvula*	4/5	8/9	C6, C8, AD1, AD3, AD6, AD10	0.0	0.0	0.0	0.3	0.5	0.9	0.0	0.1	0.1	0.1	0.0	0.1	0.6	0.2	0.2	0.0	0.1	0.7	0.1	1.8	0.0	0.0	0.1	0.0	0.0	0.0	0.0	0.0
*G. vaginalis*	3/5	3/9	C8, AD2	0.0	0.0	0.0	0.2	0.0	0.2	0.0	0.0	0.0	0.4	0.1	0.0	0.1	0.1	0.0	3.1	0.0	0.6	0.2	2.6	0.0	0.4	0.0	0.0	0.0	0.0	0.0	0.0

The table represent low abundant species (with at least 3 samples with ≥1% relative abundance and not among the 20 taxa with highest average abundance across all samples) with characteristic distribution according to control versus atopic dermatitis.

*Abbreviations*: *AD* atopic dermatitis, *C* control.

Feet were dominated by *Corynebacterium sp.* (Fig. S3). The nares were dominated by *C. propinquum and Proteobacteria sp.*, except from those dominated by *S. aureus* in AD subjects (Fig. 2).

### Changes in the mycobiome associated with AD

The bacterial domain dominated the samples of both control and AD. However, fungi were highly present at the neck of controls but not in subjects with AD (Table 2). *Malassezia globosa* was present in relative abundance ranging from 0.9–2.1% at antecubital flexures and 0.1–3,4% at the neck of healthy controls, whereas it was almost absent in AD antecubital flexures (0–0.3%) and neck (0–0.8%). The same pattern was observed for *Malasseziales sp.* (Table 2).

### Changes in virus associated with AD

**Fig. 3:**
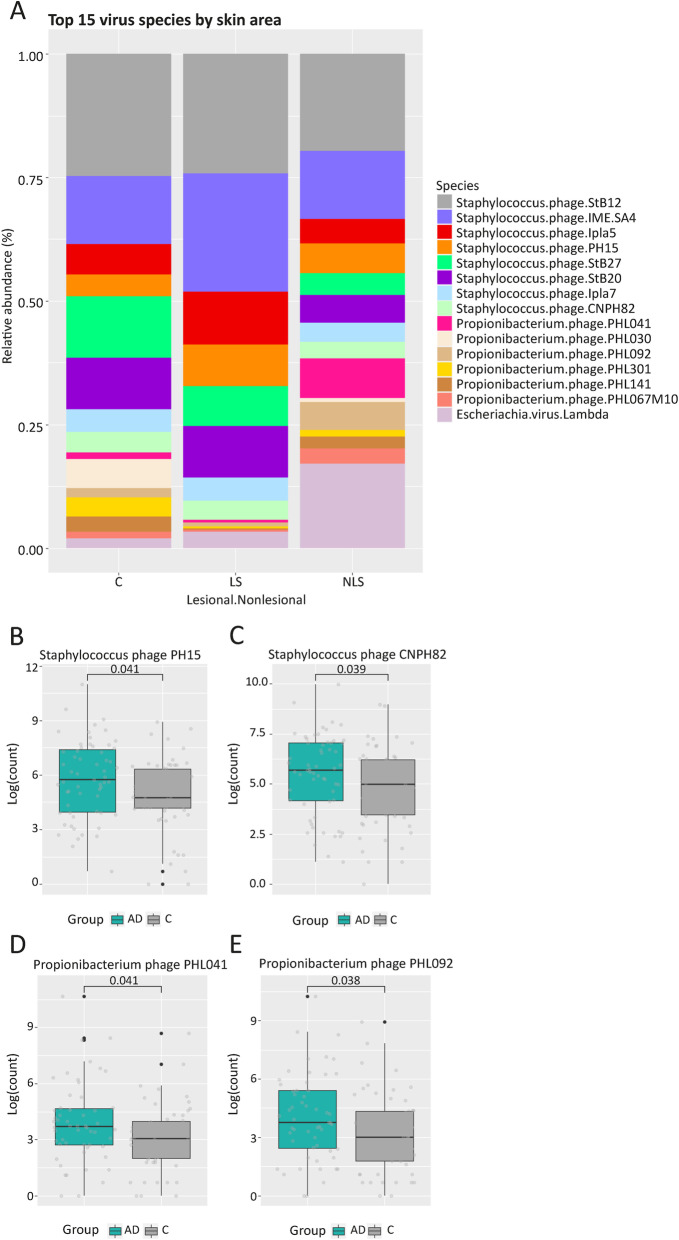
Virus in control and AD. The bar plots represent the relative abundances of the 15 most predominant viruses and show domination of *Propionibacterium* and *Staphylococcal phages*. Box plots of the non-normalized read abundances of phages with significant differences between AD and control

The number of viral reads were comparable to fungus and the *E. Coli* control had very few viral reads compared to all the skin samples (Table S2). Both absolute and relative (Fig. S5) abundance of virus (top15) were strongly dependent on the individual. Overall, *Propionibacterium* (now *Cutibacterium*) *phages* and *Staphylococcus phages* dominated the skin of both healthy controls and AD (Fig. 3). Distinct skin site related patterns appeared with more *Propionibacterium phage PHL041* in the nares and more *Staphylococcus phages* on feet (Fig. S6). In AD we found increased abundances of *Propionibacterium phages, PHL041* and *PHL092*, and *Staphylococcus epidermidis phages, CNPH82* and *PH15* [22] (Fig. 3) – not driven by subject or skin site (Fig. S7B). In lesional skin, *Staphylococcus phages* expanded (Fig. 3), including *phages Ipla5* and *Ipla7* (Fig. S7). It is also noteworthy, that the patient AD10 with extensively *S. aureus* colonization also has higher abundances of the *Stahylococcus aureus phage phiETA* (Fig. S8).

### Lesional state and *S. aureus* presence

We observed control samples grouping together while AD samples cluster further apart from each other (Fig. 1). Lesional state explained this pattern (Fig. S9A) as lesional sample composition was significantly different from control samples (PERMANOVA test; *R*^2^ = 7%, *P =* 0.0001), again with a large impact of subject on the microbial composition (*R*^2^ = 22%, *P =* 0.0001). However, testing whether the lesional versus non-lesional state explained microbial composition variance did not achieve statistical significance.

**Fig. 4:**
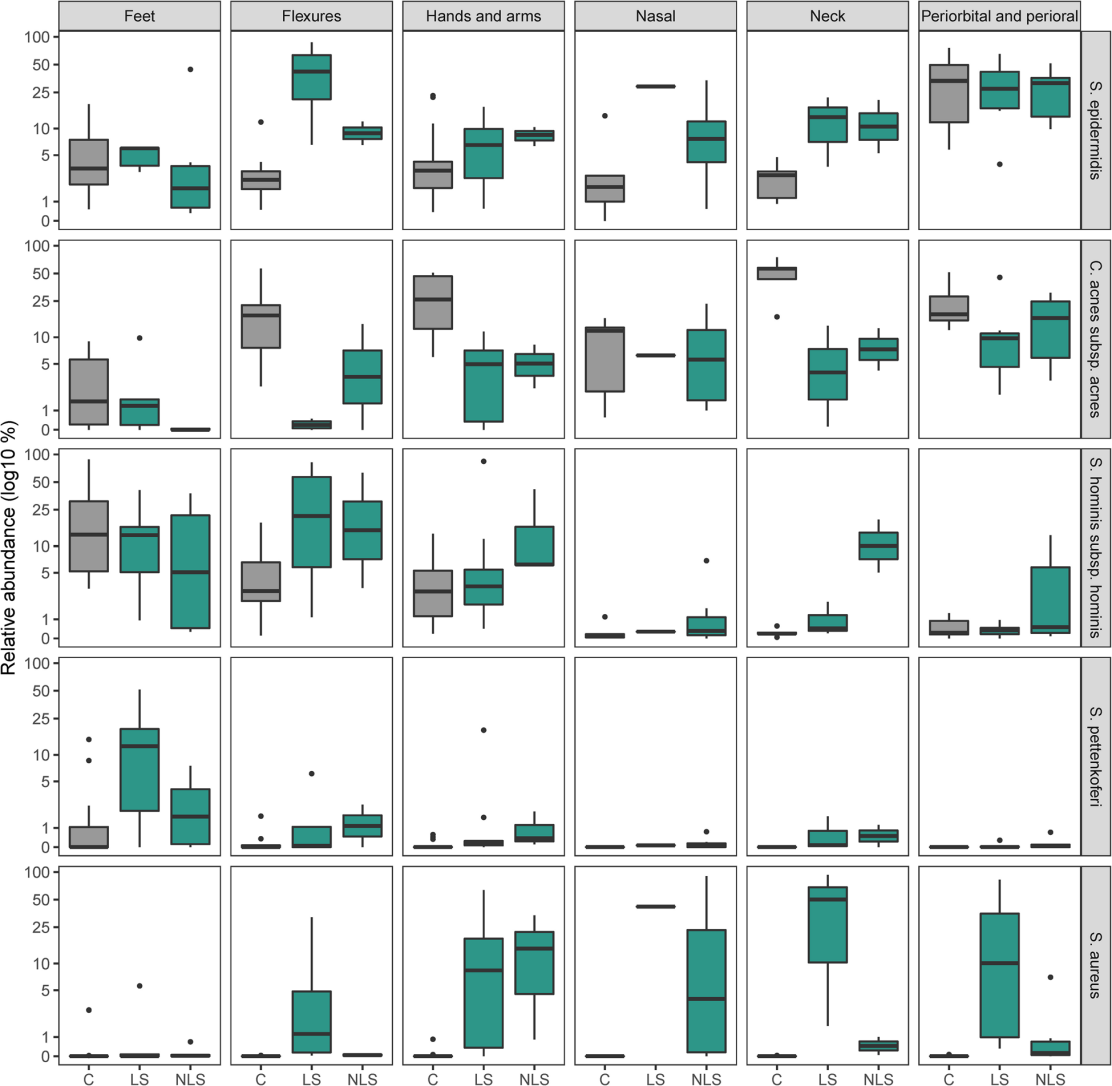
Relative abundances of Staphylococcal species and *Cutibacterium acnes.* Boxplots of mean log10 transformed relative abundances of Staphylococcal species and *Cutibacterium acnes* grouped according to healthy control and lesional status within each grouped skin site

In lesional samples, severe AD was associated with higher *S. aureus* colonization (*r =* 0.63, *P* = 0.00013), not seen in non-lesional (*r =* 0.28, *P* = 0.15) (Fig. S9B). *S. aureus* colonization were higher across all skin sites except from the feet in lesional samples (Fig. 4). When *S. aureus* colonization was high, the relative abundance of *S. hominis* and *C. acnes* were lower (Fig. 4).

In the AD flexures, bacterial diversity (Shannon diversity) was lowest at lesional sites and *S. epidermidis* colonization seemed to accompany *S. aureus* dominance, not however at other sites (Fig. 4).

### *S. aureus* strain colonization

**Fig. 5:**
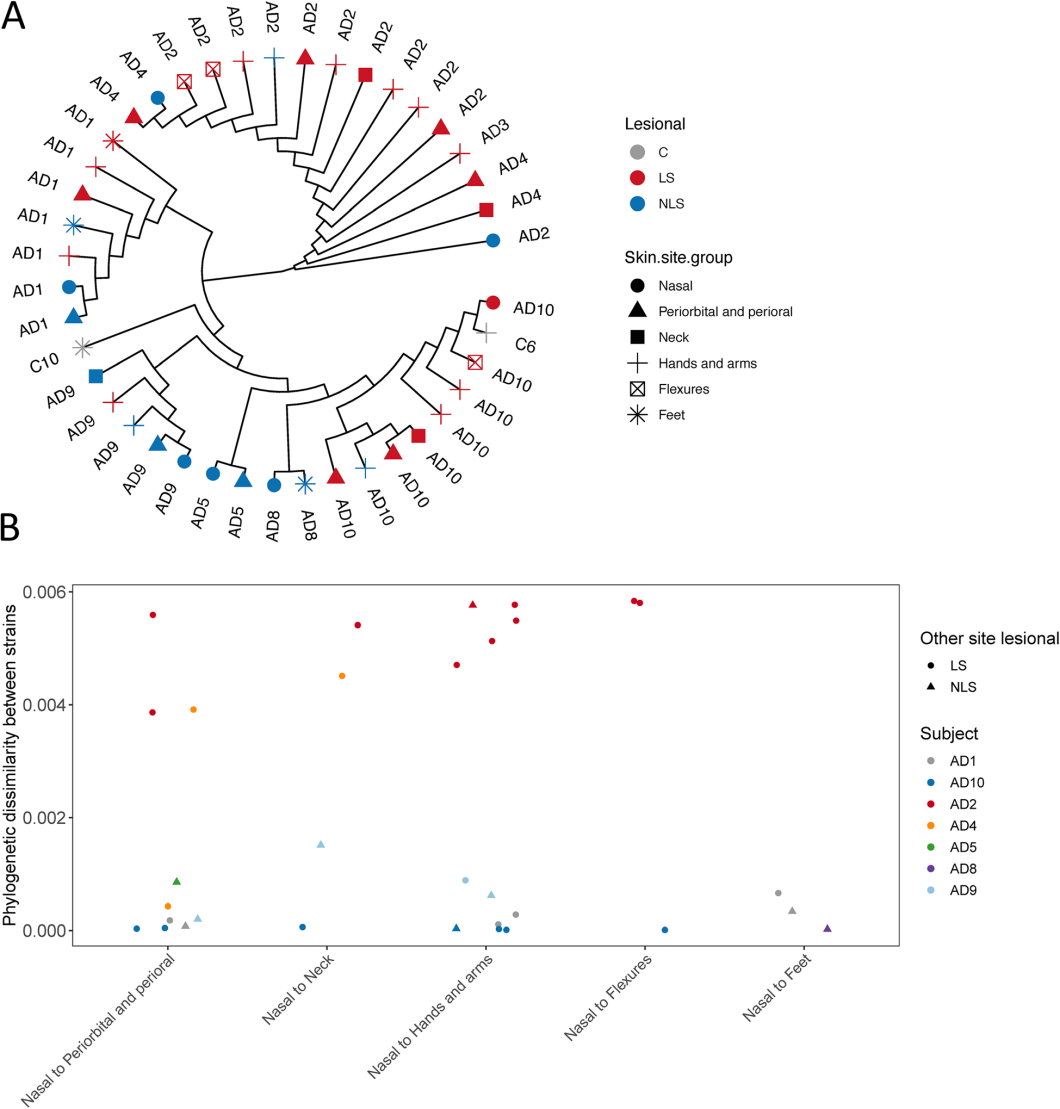
Single Nucleotide Variance analysis of *S. aureus.* The analysis was based on 3317 SNVs detected from 100 signature genes of *S. aureus* MGS.skin0051p. We detected on average 1286 SNVs per sample ranging from 313 to 2268. A) A phylogenetic tree based on *S. aureus* SNV alignments coloured and shaped by disease state and skin site. The top branch is enriched in lesional samples. B) Phylogenetic distances between nasal and other skin site samples. *S. aureus* strains are mostly subject specific

In total, 42 samples (of 121) had enough *S. aureus* coverage for single nucleotide variation (SNV) analysis, which were mostly lesional (Fig. S10). In general, the *S. aureus* strains from the same subject exhibited high similarity and lesional samples from three different AD subjects (AD2, 3 and 4) clustered together in the top branch of the tree (Fig. 5A), suggesting that the strains could be lesion and subject-specific and that different *S. aureus* strains may be implied in AD.

In analyzing the nares as reservoir of recurrent *S. aureus* infections, it could be expected that the SNVs from the nares would be similar to the ones in lesional skin, at least at the skin nearby the nose (perioral, periorbital) and at the hands (touching the nares). However, our analysis indicated no such specific pattern (Fig. 5B).

## Discussion

In this study we demonstrated a global skin dysbiosis in AD at flexures, neck, hands and arms. This is in line with findings from Baurecht and colleagues [23] showing microbial dysbiosis in AD across four skin sites (antecubital flexure, forehead, extensor- and volar forearm). The dysbiosis implicated both bacteria, fungus and virus, and especially our finding of an altered profile of bacteriophages in AD is intriguing. We also demonstrated some skin sites, feet, periorbital and perioral areas, to have more similar skin microbiome in health and AD.

We found a significantly lower alpha-diversity in AD flexures, and domination by *Staphylococcus species,* mostly *S. aureus* and *S. epidermidis*, as previously reported in the flare condition by Byrd et al. [8] In lesional samples, an increased abundance of *Staphylococcus* was accompanied by *Staphylococcus bacteriophages*, including the *Staphylococcus epidermidis phages CNPH82* and PH15. As most of these phage’s gene content are lysogenic [22], they could insert virulence factors into the bacterial genomes and contribute to a conversion from commensalism to pathogenicity. Likewise, in one patient with severe dermatitis and extensive *S. aureus* colonization, the increased abundance of the phage phi-ETA could induce more transfer of the virulence gene encoding exfoliative toxin (ET) to *S. aureus*. This toxin degrades desmosomes in the stratum granulosom [24], whereby the pathogenicity of *S. aureus* would increase and provide a competitive advantage which could lead to increased relative abundance of this bacteria. These hypotheses on potential interactions between bacteria and virus in AD would be interesting to investigate in future studies.

We also found lower relative abundances of *C. acnes* and *[P.] humerusii* in AD and a higher colonization of *Propionibacterium phages*, *PHL041* and *PHL092*. These phages might lyse *Cutibacterium* ([*Propionibacterium*]) and result in the lower relative abundance. *[P.] humerusii* [25] is a common inhabitant of the pilosebaceous unit [26], but to our knowledge this is the first study to report a difference in abundance in control versus skin disease. *C. acnes* has previously been reported to be reduced in AD skin [14, 27, 28]. It is a lipophilic bacteria, and altered sources of fatty acid substrates in AD skin [23, 29] might also restrict its growth. *C. acnes* ferments glycerol into short-chain fatty acids, including propionic acid, which can inhibit growth of *S. aureus* [30].

*M. luteus* was more abundant particularly in two AD subjects and may indicate a certain AD dermotype, as recently suggested [14]. *M. luteus* has the capability to augment proliferation of virulence of *S. aureus* [31]. The role of *M. luteus* in AD should be investigated further in future studies. A new important finding of this study is a potential association between *M. osloensis* and AD*. Moraxella* species are part of the human skin microbiota [32] and *M. osloensis* is a rare causative organism of human infections [33–37]. It may therefore be relevant to investigate further whether *M. osloensis* is an active player in AD.

No study has yet characterized the skin microbiome of the anterior triangle of the neck in AD, which is colonized with high amount of Staphylococcal species, but interestingly, also characterized by a lack of *Malassezia* species. *Malassezia* is a genus of lipophilic yeasts and comprises the most common fungi on healthy human skin [38]. The role of *Malassezia* in AD is debated. It is often attributed a pathogenic role. Especially in a subset of AD patients with symptoms predominating on the head and neck. However, despite that numerous studies have attempted to show a difference in frequency of *Malassezia* skin colonization in AD patients, there is no such evidence (reviewed by Glatz et al. [38] and Tsakok et al. [39]). As some randomized controlled studies report beneficial effects of anti-fungal treatment [39], we asked the patients whether they have used antifungal treatment (Table 1) and 2/5 might have used Nizoral shampoo around study participation, which could explain some lack of *Malassezia* in AD, but not in all patients. However, two recent microbiome studies indicate a lack of *Malassezia* in AD too [40, 41] – with one of the studies conducted in an AD prone population, with past AD episodes [40], thus not expected to use antifungal treatment. Poor growth conditions in dry AD skin and absence of *C. acnes* providing substrates for *Malassezia* could restrict the growth.

Variability in beta-diversity within AD sites are higher than in controls, which we ascribe differences in lesional state. Other endogenous and exogenous factors might also explain larger variability in AD samples. Clinically the disease shows great patient to patient variability, and effort are being put into defining endotypes of the disease [10, 14, 42, 43]. It was recently reported that lesional AD skin is characterized by larger inter- and intra-patient microbiome variability than non-lesional skin [44]. The inter-patient variability mainly originated from *S. aureus* abundance.

Here, lesional samples were characterized by higher *S. aureus* colonization across all skin sites, except from the feet. We find that high abundance of *S. aureus* was accompanied by lower relative abundances of *S. hominis,* which is in line with data from Baurecht et al. showing decreased *S. hominis* at four AD skin sites [23]. Nakatsuji et al. reports that AD patients lack strains of coagulase-negative *Staphylococcus (including S. hominis* strains) producing antimicrobial peptides against *S. aureus* [21], which can explain their opposing presence in the skin microbiome. In a previous study, reintroducing antimicrobial coagulase negative strains to human subjects with AD decreased *S. aureus* colonization [21]. Other studies have also succeeded in treating AD with microorganisms [19, 20], indicating that microbial transplants could be a promising strategy in AD management and highlighting the clinical relevance of finding skin site-specific species. Our data furthermore indicate that it is highly relevant to investigate both bacteria, fungi and virus for understanding skin dysbiosis. Using phages for targeting microbial dysbiosis in AD yields potential, which is supported by the specificity of phages [45]. Phage-derived endolysins have been used to target *S. aureus* specifically, however not in AD patients [46].

### Strengths and limitations

Published skin shotgun sequencing data from AD is sparse and having 121 samples successfully analyzed is a large number. However, a substantial number of samples failed sequencing due to insufficient biomass, making it difficult to evaluate the influence of all relevant factors. The low biomass is a known challenge [6, 47]. We included AD patients in systemic treatment, which may affect the microbiome. However, even though the patients using topicals were instructed not to apply it 48 h before, we did not found differences in microbial composition between AD patients in topical versus systemic treatment (PERMANOVA, *R*^2^ = 4%, *P* = 0.98). Another limitation is the use of DNA to characterize skin microbiota as we cannot assess if the microbes are dead or alive or metabolically active. It is also difficult to analyze both bacteria, fungus and virus in the same dataset and it should be underlined that the DNA extraction protocol was optimized for bacteria. It is uncertain if the viral reads come from a phage or phage DNA inserted into a bacterial genome. Reference databases lack annotation for some organisms, which is the case of *Malassezia restricta* in this study. Studies combining microbiome and transcriptome data in AD are emerging [6, 10] and in general, future studies would benefit from integrating omics data in capturing the flow of information underlying disease states in AD.

## Conclusion

Though the microbial dysbiosis in AD is global, some sites are more affected than others. In our study, the flexures and neck showed marked taxonomical changes compared to healthy control. The flexures with lower alpha-diversity and high *S. aureus* abundance and high abundance of *S. epidermidis* in lesions, while at the neck *Malassezia* species were not detected. *S. aureus* colonization was observed across all lesional skin sites except the feet. In general, the *S. aureus* strains were highly similar within subjects both between lesional and non-lesional samples, indicating that more *S. aureus* strains are involved in AD. *S. aureus* may outgrow the coagulase negative *S. hominis* and *C. acnes.* Furthermore, phages targeted [*Propionibacterium]* and virulent phages such as *Staphylococcus phi-ETA phage* might support the growth of *S. aureus. M. luteus and M. osloensis* are more abundant in AD and may be active players in the disease.

## Methods

### Study participants

Samples from 10 adult patients with current atopic- and hand dermatitis and 5 healthy age and sex matched controls were enrolled from March to July 2018. All patients were recruited from the Department of Dermatology and Allergy at Herlev and Gentofte Hospital, Denmark. AD had been diagnosed by a physician and confirmed by the UK Working Party Diagnostic Criteria [48] at inclusion. Patients were characterized by demographic data, treatment, co-morbidities, FLG mutations (R501X, 2282del4, and R2447X) when available in their medical records and disease severity assessed by SCORAD and Hand Eczema Severity Index (HECSI) (Table 1). Exclusion criteria included active infections, use of antibiotics or probiotics within the past 4 weeks and for healthy volunteers a history of eczema. Two days before sampling, subjects were instructed not to shower or use topicals.

### Sampling, DNA extraction and sequencing

Skin samples were collected using eSwabs from non-overlapping areas on 14 sites (Table 1) as described previously [49]. When eczema was present, the area affected, and morphology were described (Table S1).

DNA was extracted using QIAamp DNA Microbiome Kit (QIAGEN, lot no.: 154026306) according to manufactures’ protocol. The DNA was randomly sheared into fragments of around 350 basepairs by sonication. Library preparation was performed with the NEBNext Ultra II Library Prep Kit for Illumina (New England Biolabs). Paired-end sequencing (2 × 150 basepair) was performed on an Illumina platform.

### Preprocessing of sequencing data and mapping reads to the gene catalog

For analyses of the bacteriome and mycobiome, adaptor removal from raw FASTQ files was performed using KneadData (v. 0.6.1) and Trimmomatic. Trimmed reads shorter than 100 bases were discarded. PCR/optical duplicates were removed using samtools (v. 1.6). Host reads mapping to the human reference genome GRCh38 (with Bowtie2 v. 0.0.3.2) were excluded. Read pairs in which both reads passed filtering were retained and mapped using BWA mem (v 0.7.16a) to a reference gene catalogue built by Clinical Microbiomics from shotgun sequencing data from 1972 skin microbiome samples, containing 4.4 million non-redundant genes and 234 skin-associated metagenomic species (MGS, v3.0) [50] with highly coherent gene abundance and base composition have been identified (as described in Nielsen et al.).

To taxonomically annotate the MGSs, all the catalog genes has been blasted to the NCBI RefSeq genome database (2019-02-18). A MGS was considered detected if read pairs were mapped to at least three of its 100 signature genes. Normalization was done to the effective gene length and then to sum 100%, resulting in a relative abundance estimate of each MGS.

For analyses of virus, quality processed FASTQ files (AdaptorRemoval-2.1.3) were assigned taxonomic labels using Kraken 2.

### Ultrahigh-resolution phylogenetic profiling

For all samples in which *S. aureus* (MGS.skin0051p) was detected, we extracted the reads aligning to 100 signature genes of MGS.skin0051p and used the BCFtools (v. 1.6) multiallelic genotype caller to summarize the counts of each base observed in each position (requiring: sequencing depth ≥ 5 and ≥ 80% major allele fraction and filtering to remove indels and SNVs near indels). Samples with at least 40% of the positions with a called base were retained for further analysis.

Maximum-Likelihood phylogenetic trees with pairwise distances were inferred using IQ-TREE (v. 1.6) based on the alignment of the Single Nucleotide Variants (SNV) considered from the 100 signature genes for MGS.skin0051p. By using ModelFinder Plus we selected the best substitution model estimated separately for each gene. This resulted in phylogenetic trees where each branch represents the most dominant *S. aureus* strain in a given sample. The phylogenetic distances matrix was constructed from all pairwise tree-branch length distances between any two samples in the tree (i.e. patristic pairwise distances).

### Statistical analyses

Descriptive data on relative abundances was both analysed according to individuals and disease groups. Beta-diversity was estimated by Bray-Curtis dissimilarity among samples and alpha-diversity using Shannon’s index, both measures were based on MGS abundances. Permutational multivariate analysis of variance (PERMANOVA) was used to assess the effects of disease (AD vs Control) or lesional state (Control, Lesional and Non-lesional), considering a nested model of disease within skin area and adjusting for subject variability. Pearson correlations were calculated between AD severity scores and *S. aureus* abundance. Wilcoxon signed-rank test was used to compare viral abundances between two groups. Outliers in box plots were defined by the interquartile range rule. Visualizations and statistics were conducted in R (R core team, version 4.0.4, http://www.R-project.org/), where we also used the application Pavian for gathering Kraken reports.

## Supplementary Information

**Additional file 1.**

## Data Availability

The sequencing datasets generated and analysed during the study are not publicly available due to privacy of the participants but are available from the corresponding author upon reasonable request.

## Abbreviations

AD: Atopic dermatitis
*S. aureus*: *Staphylococcus aureus*
*S. epidermidis*: *Staphylococcus epidermidis*
*S. hominis*: *Staphylococcus hominis*
SCORAD: Severity Scoring of Atopic Dermatitis
PERMANOVA: Permutational multivariate analysis of variance
PCoA: Principal coordinate analysis
*E. coli*: *Escherichia coli*
*S. saccharolyticus*: *Staphylococcus saccharolyticus*
*S. lugdunensis*: *Staphylococcus lugdunensis*
*C. acnes*: *Cutibacterium acnes*
SNV: Single nucleotide variation
*[P.] humerusii*: *[Propionibacterium] humerusii*
*M. luteus*: *Micrococcus luteus*
*M. osloensis*: *Moraxella osloensis.*
