# Supplementary material for: Skin dysbiosis in the microbiome in atopic dermatitis is site-specific and involves bacteria, fungus and virus
Source: BMC Microbiol. 2021 Sep 23;21:256. doi: 10.1186/s12866-021-02302-2 (PMC8459459; doi:10.1186/s12866-021-02302-2)
Supplement: Supplementary file 1 — Additional file 1. [file 12866_2021_2302_MOESM1_ESM.docx]

**Electronic Supplementary Material**

This supplementary material has been provided by the authors to give readers additional information about their work.

Supplement to: RD Bjerre^1*^, JB Holm^2^, A Palleja^2^, J Sølberg^1^, L Skov^3^, JD Johansen^1^. Skin dysbiosis in the microbiome in atopic dermatitis is site-specific and involves bacteria, fungus and virus.

*Corresponding author: rie.dybboe.bjerre@regionh.dk

Affiliations:

*^1^ National Allergy Research Centre, Herlev and Gentofte Hospital, University of Copenhagen, Denmark*

*^2^ Clinical Microbiomics, Fruebjergvej 3, 2100 Copenhagen, Denmark
^3^ Department of Dermatology and Allergy, Herlev and Gentofte Hospital, University of Copenhagen, Denmark*

| **Sample no** | **Area affected (%)** | | | | **Morphology** | | | | | |
| --- | --- | --- | --- | --- | --- | --- | --- | --- | --- | --- |
|  | **1-25** | **26-50** | **51-75** | **76-100** | **Erythema** | **Edema** | **Lichenification** | **Excoriation** | **Dryness** | **Fissures** |
| 2.3 | x |  |  |  | x |  | x |  |  |  |
| 2.9 | x |  |  |  | x | x | x |  | x |  |
| 2.10 |  |  | x |  | x |  |  |  | x |  |
| 2.12 | x |  |  |  | x |  | x |  |  |  |
| 3.2 |  |  | x |  | x | x | x |  |  |  |
| 3.3 |  |  |  | x | x |  | x |  | x | X |
| 3.4 |  | x |  |  | x |  |  |  |  |  |
| 3.5 |  | x |  |  | x | x |  |  |  |  |
| 3.6 |  | x |  |  | x |  |  | x |  |  |
| 3.7 |  |  | x |  | x | x |  |  |  |  |
| 3.8 |  | x |  |  | x | x |  |  |  | X |
| 3.10 | x |  |  |  | x |  |  |  |  |  |
| 3.11 | x |  |  |  | x | x |  |  |  |  |
| 4.10 | x |  |  |  | x |  |  |  | x |  |
| 5.2 | x |  |  |  |  |  | x |  | x |  |
| 5.3 | x |  |  |  |  |  | x |  | x |  |
| 5.4 | x |  |  |  |  |  |  |  | x |  |
| 5.9 | x |  |  |  | x | x |  |  | x |  |
| 5.14 | x |  |  |  |  |  |  |  | x |  |
| 8.14 | x |  |  |  | x |  | x |  | x |  |
| 18.5 | x |  |  |  | x |  | x |  | x |  |
| 18.6 | x |  |  |  | x |  |  |  |  |  |
| 18.10 | x |  |  |  |  |  |  |  | x |  |
| 18.12 | x |  |  |  |  |  | x |  | x |  |
| 18.13 | x |  |  |  |  |  |  |  | x |  |
| 20.1 | x |  |  |  | x |  | x | x |  |  |
| 20.2 |  | x |  |  | x | x | x | x |  |  |
| 20.3 |  | x |  |  | x |  | x | x |  |  |
| 20.4 |  | x |  |  | x | x | x | x |  |  |
| 20.6 |  | x |  |  | x | x | x |  | x |  |
| 20.7 |  | x |  |  | x | x | x |  | x |  |
| 20.10 | x |  |  |  | x |  |  |  | x | x |

**Table S1: Clinical description of lesional samples**

**Table S2: Classification report from Kraken 2**

**Figure S1: Bray-Curtis Dissimilarity between controls and patients with AD**

Skin sites are shown at the x-axis.


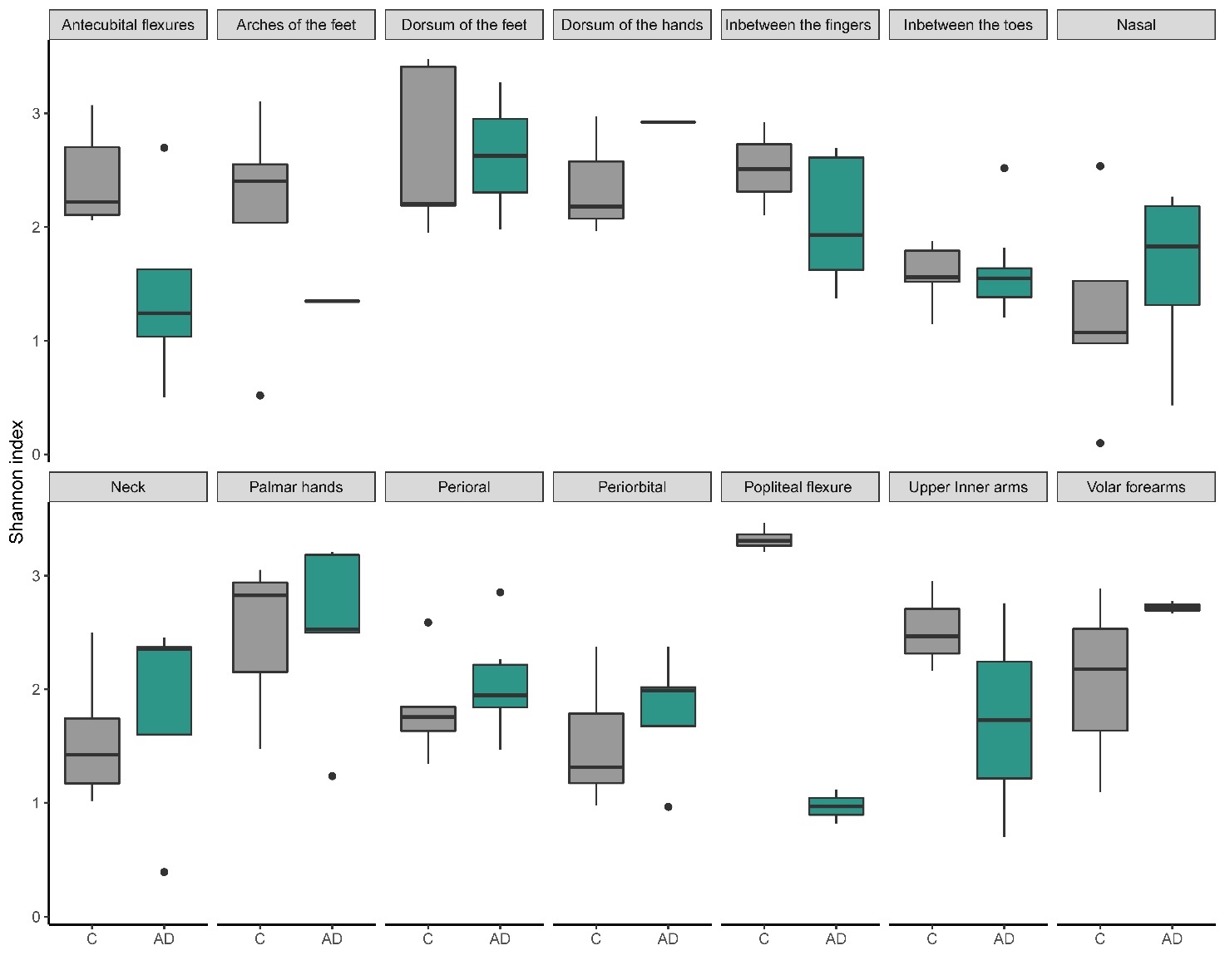


**Figure S2: Lower bacterial diversity at the flexures in AD.**

Boxplots of Shannon diversity grouped according to healthy control (C) and AD samples within each skin site.

**Figure S3: Stacked bar plots of relative abundances of bacterial species at different skin sites in healthy controls and patients with AD.** Sample numbers are shown at the x-axis where the first number refers to the subject (inclusion number) and the second the skin site sampled. The figure shows the 20 taxa with highest average abundance across all samples. The taxa are sorted from most abundant to least abundant.

**Figure S4: Stacked par plots of relative abundances of bacterial species arranged according to individual**. Skin sites are shown at the x-axis. The figure shows the 20 taxa with highest average abundance across all samples. The taxa are sorted from most abundant to least abundant.


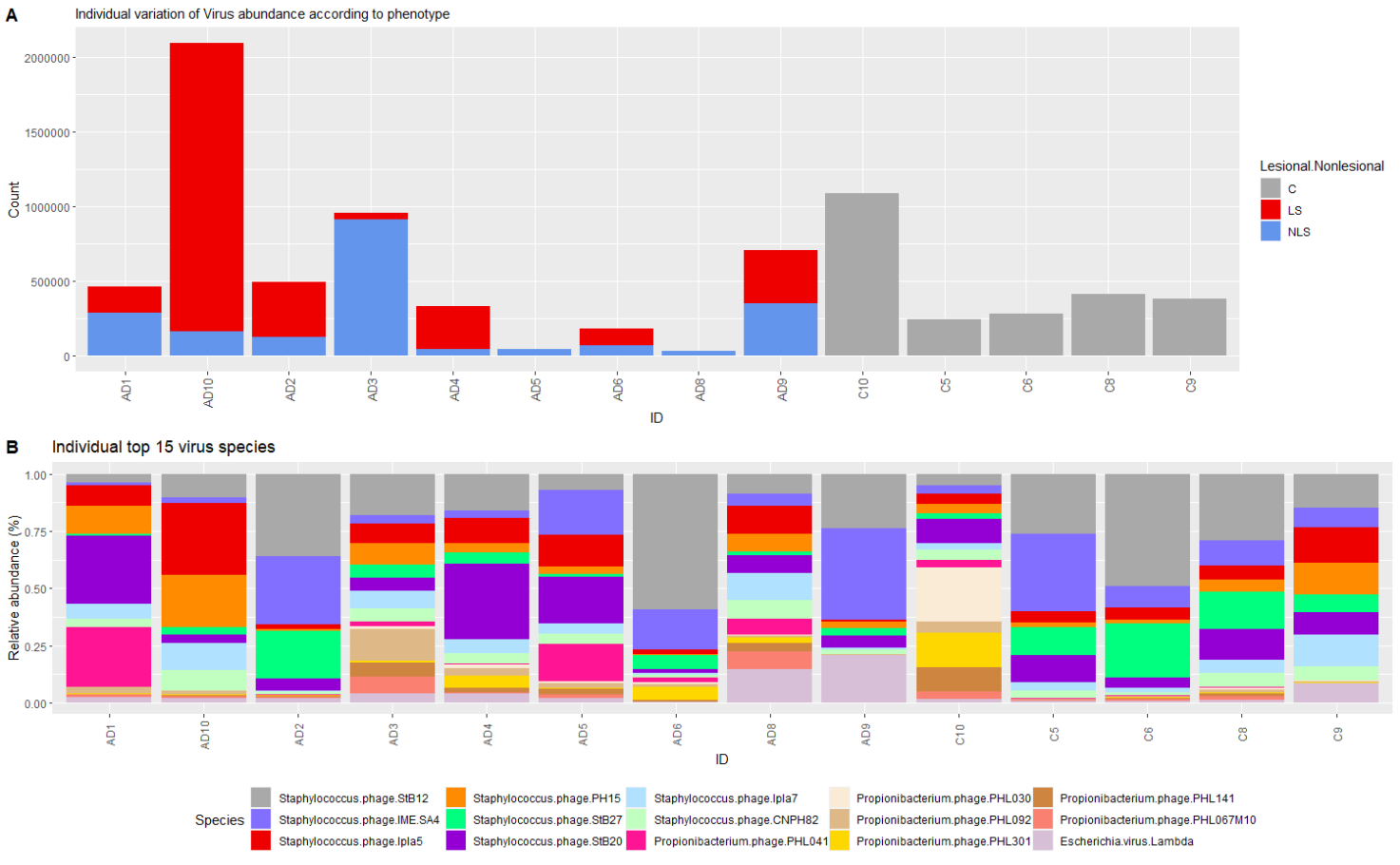


**Figure S5: Individual variation in total and relative viral abundances**

Individuals are shown on the x-axis. The absolute number of reads (A) are divided according to lesional state (LS: Lesional, NLS: Nonlesional, C: Control). Figure B) shows the 15 viruses with highest relative abundance across all samples.


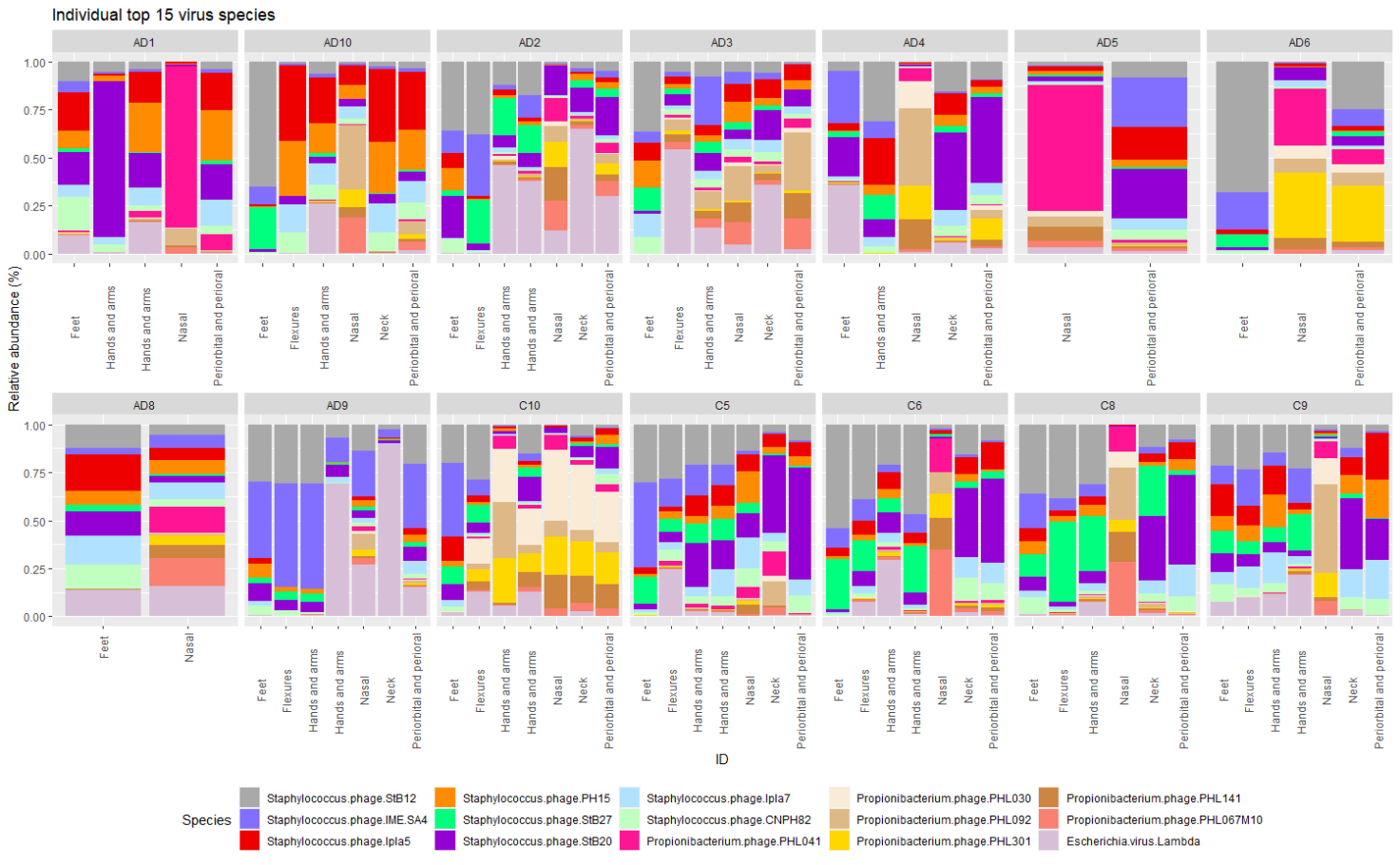


**Figure S6:** **Stacked par plots of relative abundances of viruses grouped according to individual**.

Skin groups are shown at the x-axis. The figure shows the 15 viruses with highest relative abundance across all samples.


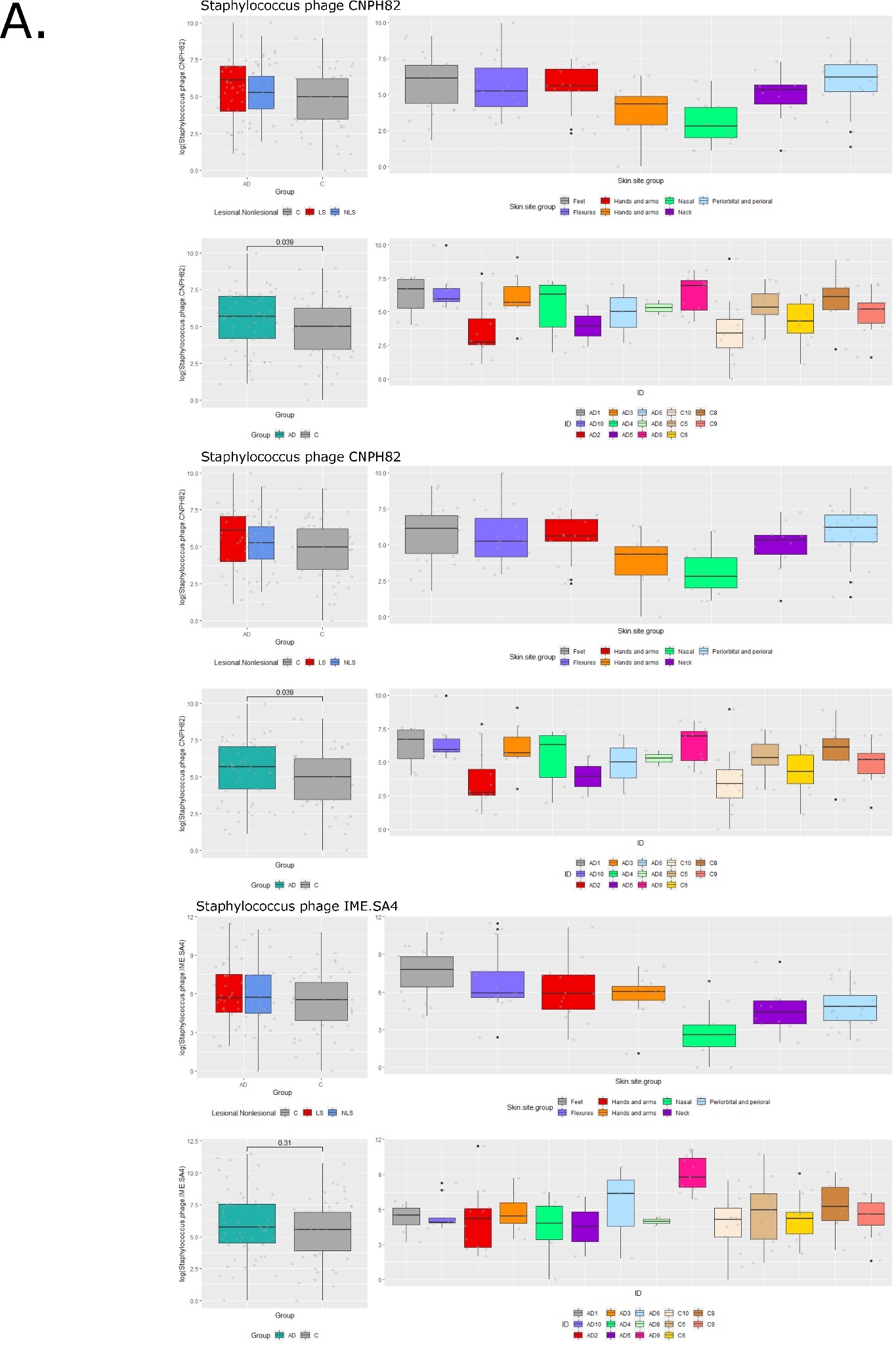


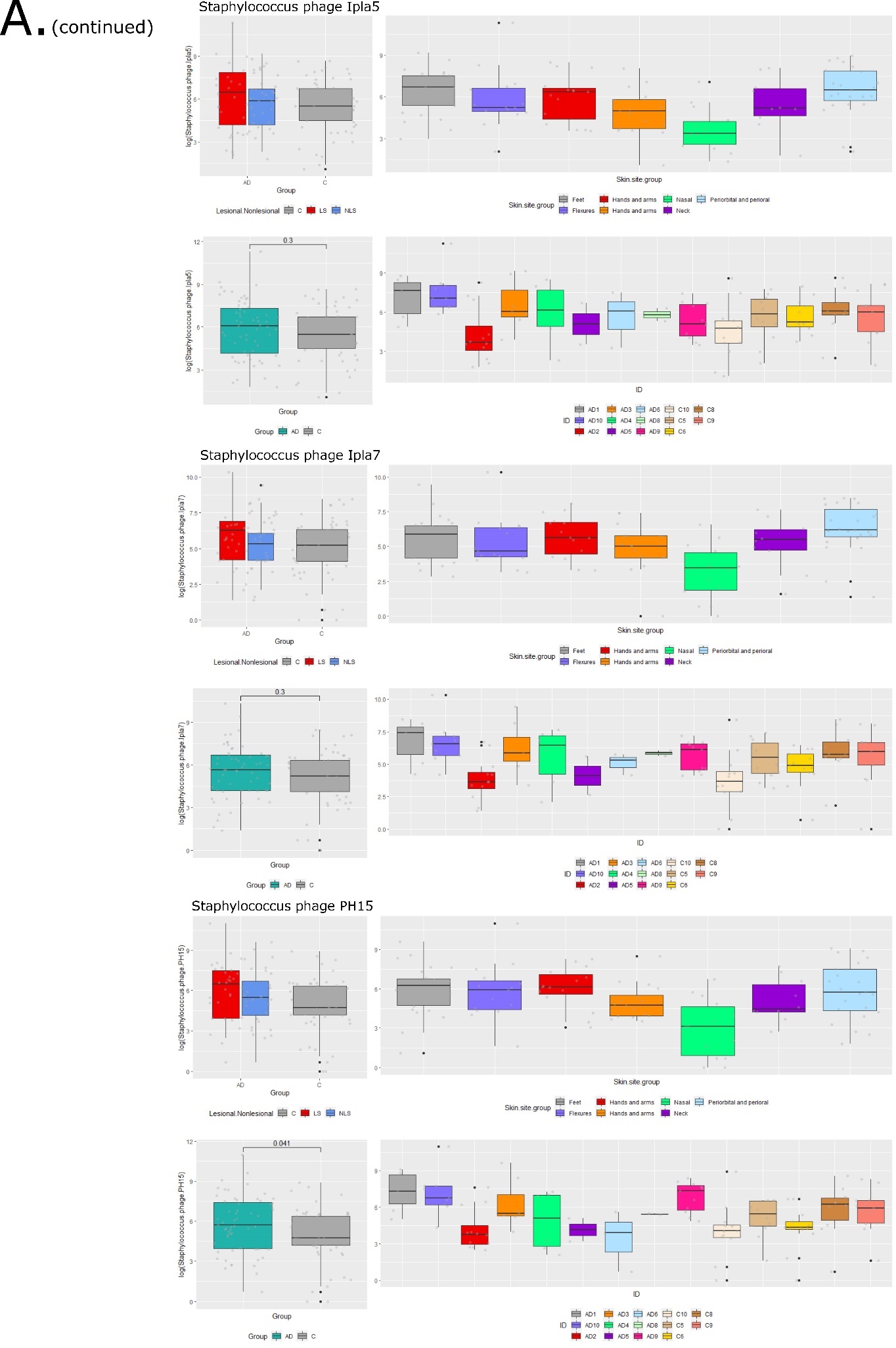


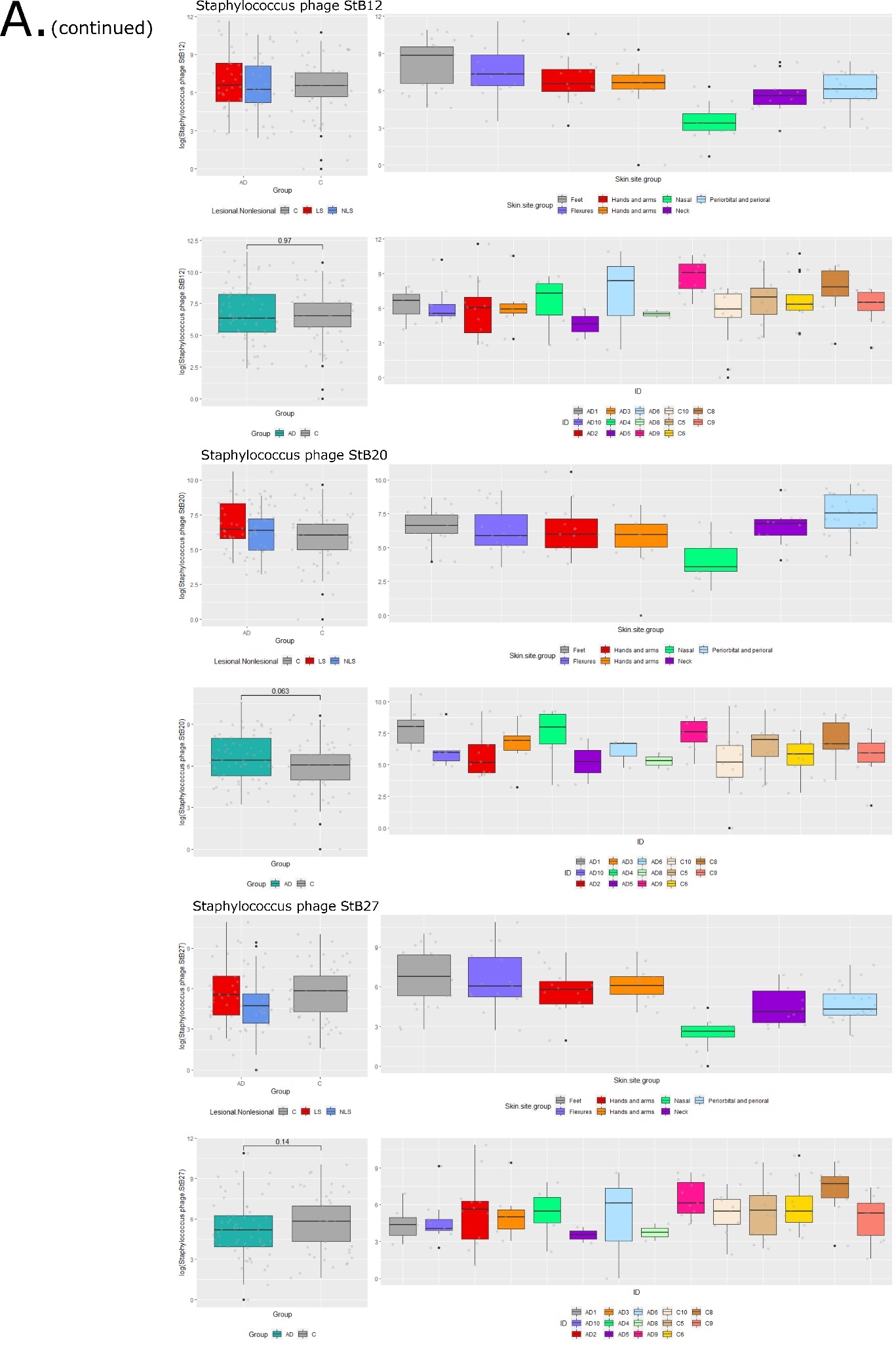


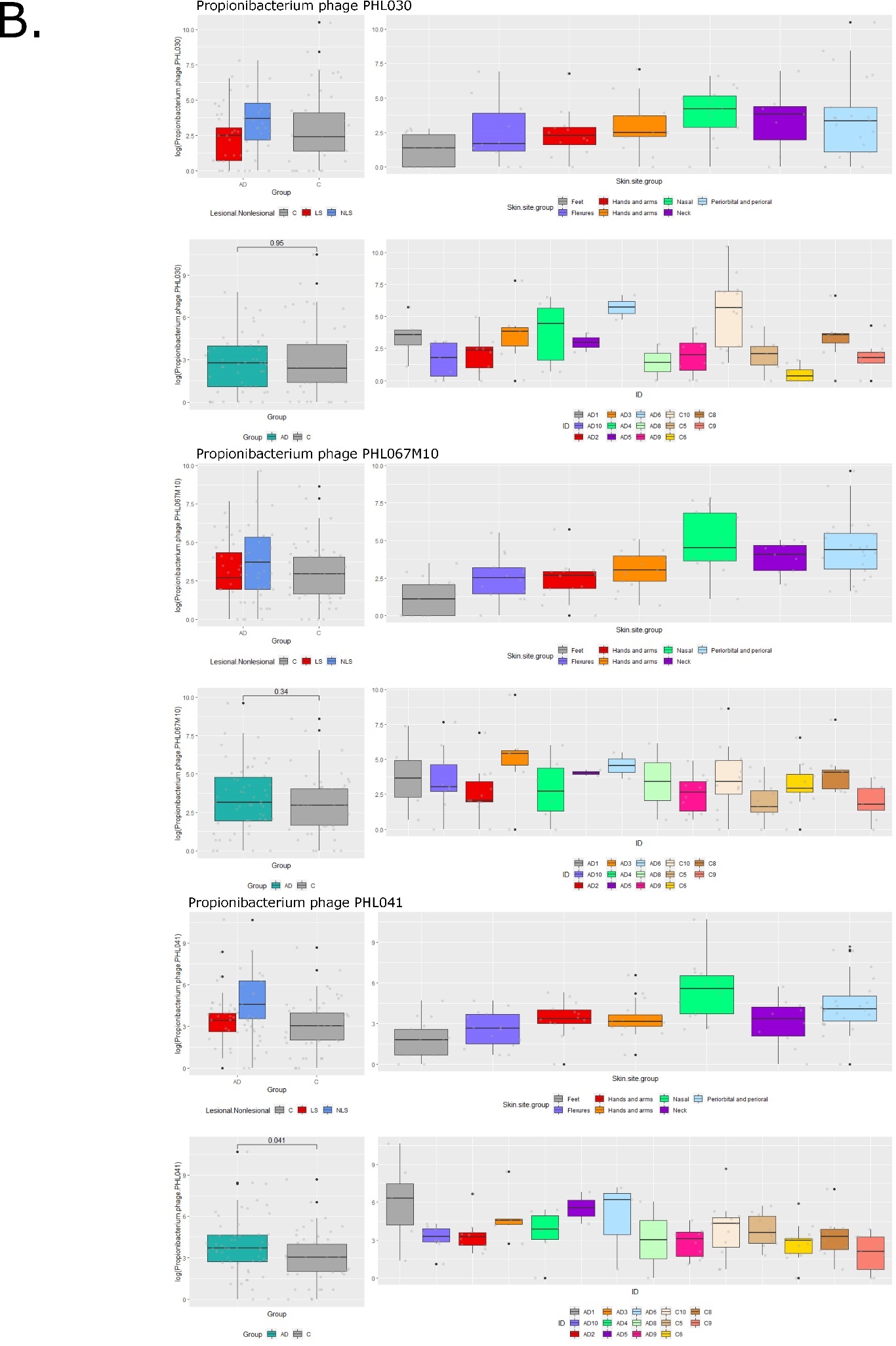


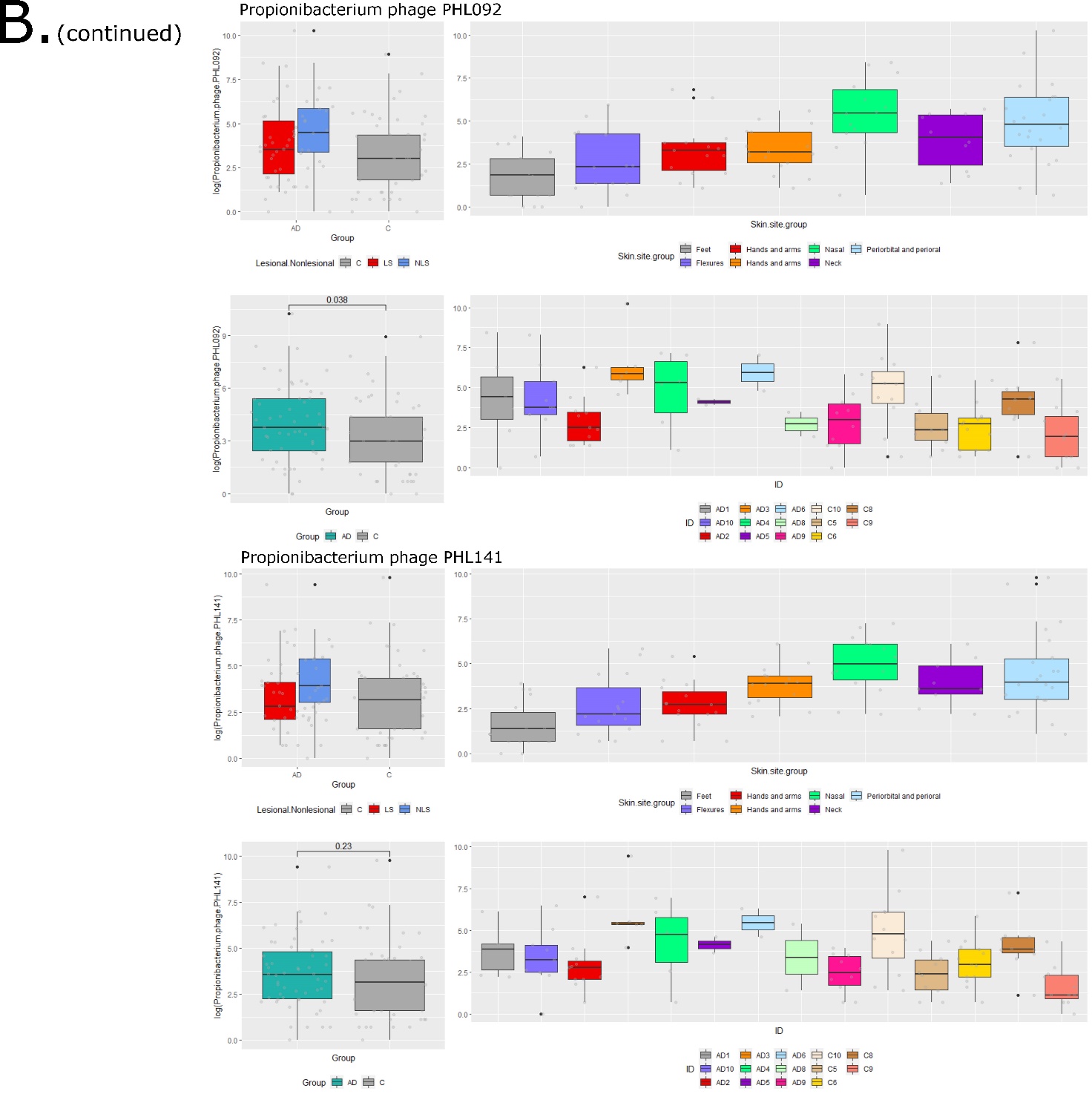


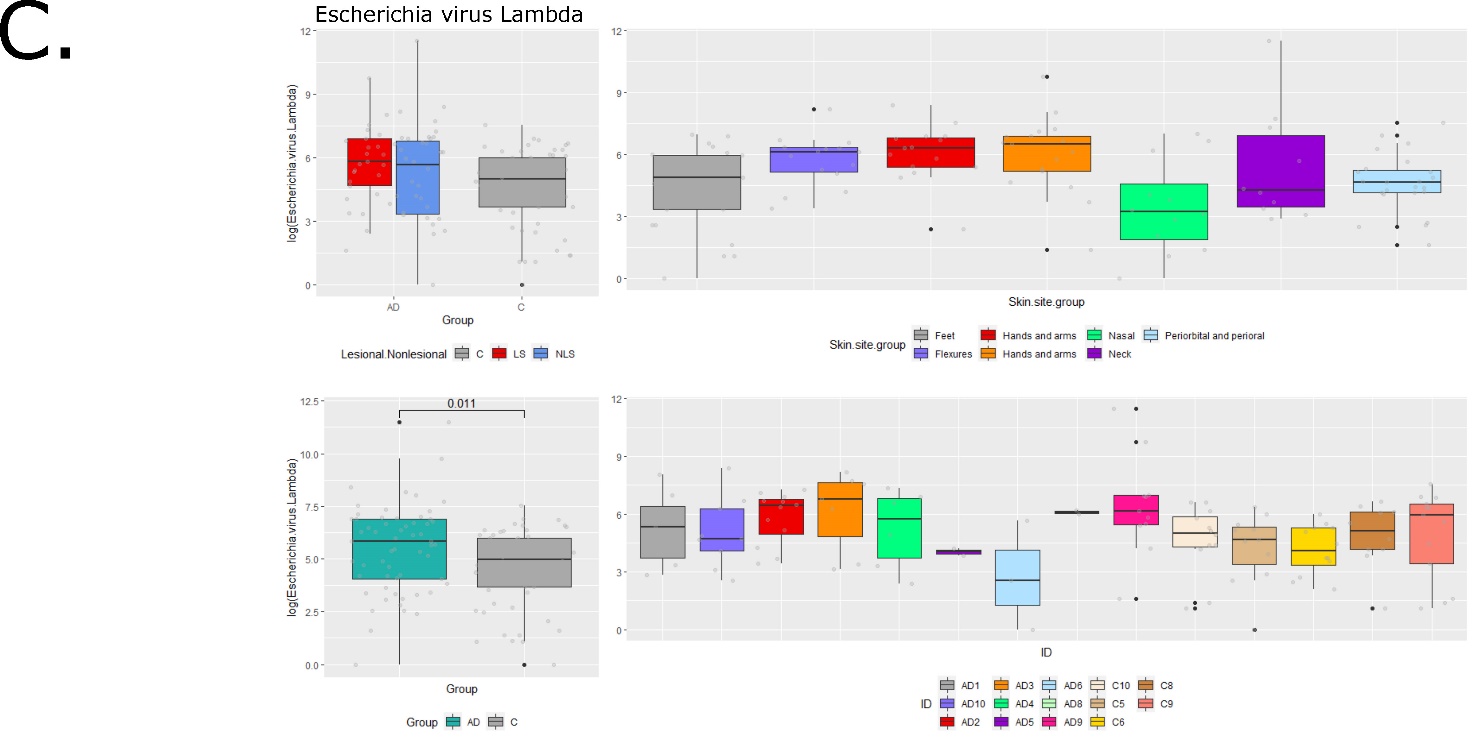


**Figure S7: Boxplots of the top 15 most abundant viruses**

The non-nomalized read abundances (counts) of A) *Staphylococcus phages*, B) *Propionibacterium phages* and C) *Escherichia lambda phage* are grouped according healthy control (C) and AD, including lesional state (LS:Lesional, NLS:Nonlesional, C:Control) to the left. To the right groupings are according to skin site group and individual.


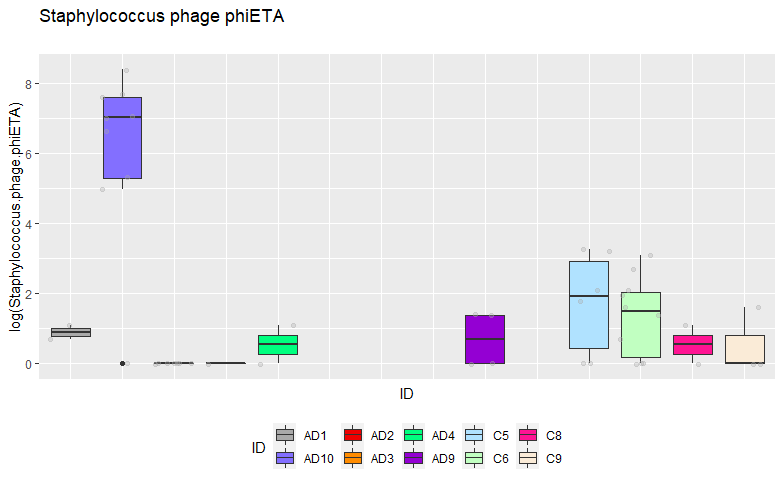


**Figure S8: Boxplot of the non-normalized read abundance of *Staphylococcus phage phiETA***

Grouped according to individual.

**A)**


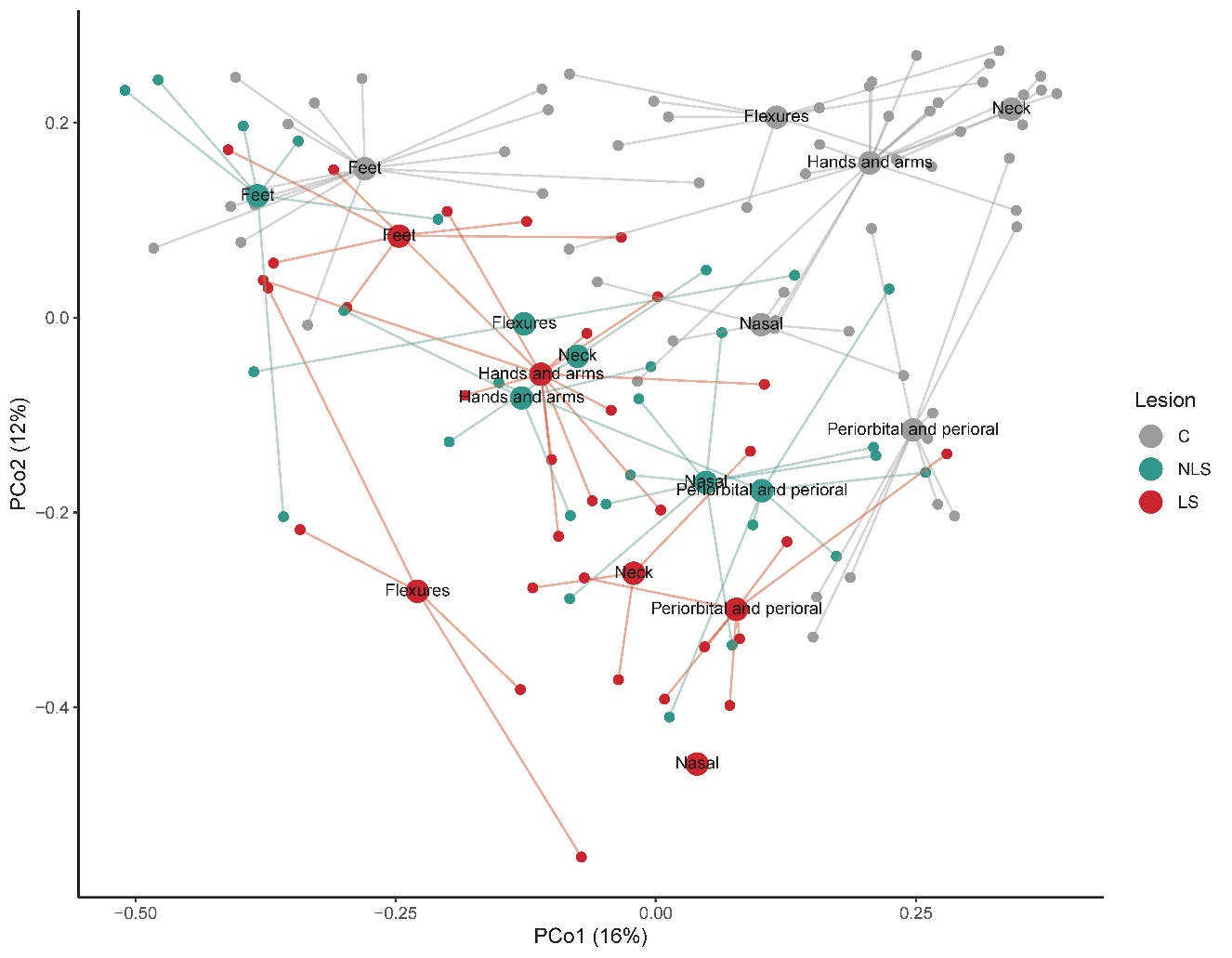


**B)**


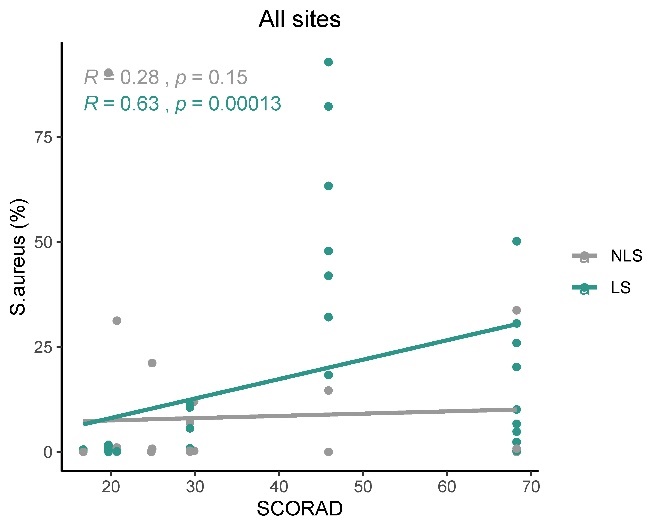


**Figure S9: Lesional state, effects on clustering according to skin site and *S. aureus* colonization**

The 6 skin area groups are: Feet (arches of the feet, dorsum of the feet and between the toes; C N=15, AD N=11), flexures (antecubital and popliteal flexures; C N=9, AD N=6), hands and arms (palmar and dorsum of the hands, between the fingers, volar forearms and upper inner arms; C N=17, AD N=15), Nasal (C N=5, AD N=9), neck (C N=5, AD N=5), and periorbital and perioral (C N=10, AD N=13).

A, Principal coordinate analysis based on Bray-Curtis distances between samples. Centroids represent the arithmetic mean position of the points belonging to the specific category.

B, Proportion *Staphylococcus aureus* versus SCORAD for all skin sites of AD patients according to lesional state, Pearson partial correlation.

**
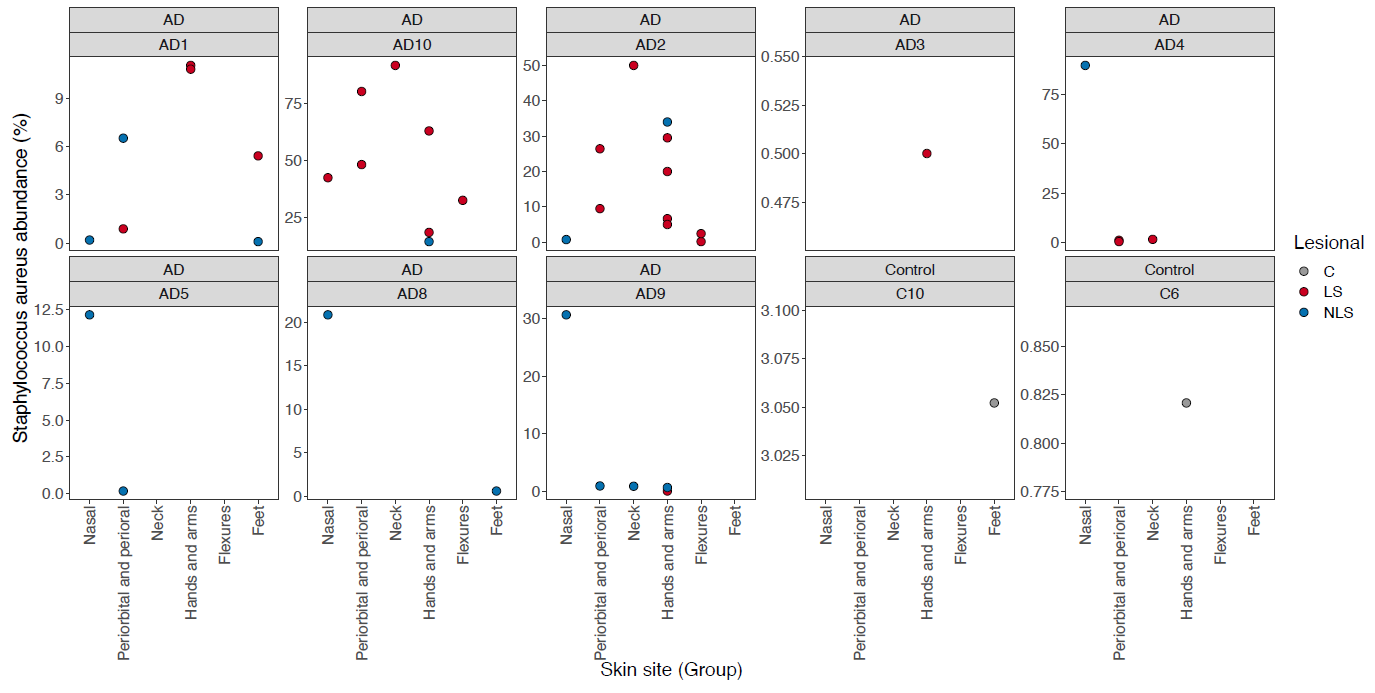
**

**Figure S10: Samples with *S. aureus* detected with enough SNV coverage (N=42)**

Skin site is shown at the x-axis, *S. aureus* abundance on the y-axis coloured according to disease state.

**Figure S11: Effect of subject**

Principal coordinate analysis based on Bray-Curtis distances between samples. Centroids represent the arithmetic mean position of the points belonging to the specific category.
